# Network proximity analysis as a theoretical model for identifying potential novel therapies in primary sclerosing cholangitis

**DOI:** 10.1186/s12920-024-01927-2

**Published:** 2024-06-11

**Authors:** Jessica Leighton, David E. J. Jones, Jessica K. Dyson, Heather J. Cordell

**Affiliations:** 1 Translational and Clinical Research Institute, Newcastle University, Newcastle upon Tyne, UK; 2 Liver Unit, Freeman Hospital, Newcastle upon Tyne Hospitals NHS Foundation Trust, Newcastle upon Tyne, UK; 3 Population Health Sciences Institute, Faculty of Medical Sciences, Newcastle University, Newcastle upon Tyne, UK

**Keywords:** Network proximity analysis, Genome-wide association studies, Therapeutics, Autoimmune liver disease, Primary sclerosing cholangitis, Hepatology, Druggable genome

## Abstract

Primary Sclerosing Cholangitis (PSC) is a progressive cholestatic liver disease with no licensed therapies. Previous Genome Wide Association Studies (GWAS) have identified genes that correlate significantly with PSC, and these were identified by systematic review. Here we use novel Network Proximity Analysis (NPA) methods to identify already licensed candidate drugs that may have an effect on the genetically coded aspects of PSC pathophysiology.

Over 2000 agents were identified as significantly linked to genes implicated in PSC by this method. The most significant results include previously researched agents such as metronidazole, as well as biological agents such as basiliximab, abatacept and belatacept. This *in silico* analysis could potentially serve as a basis for developing novel clinical trials in this rare disease.

**Supplementary Information:**

The online version contains supplementary material available at 10.1186/s12920-024-01927-2.

## Introduction

Primary Sclerosing Cholangitis (PSC) is a rare, progressive cholestatic autoimmune liver disease that leads to chronic liver injury, biliary cirrhosis (as a consequence of the development of significant biliary stricturing) with its associated complications of bacterial cholangitis, and cholangiocarcinoma [1]. PSC patients also experience cholestatic disease symptoms, including pruritus and fatigue. At present there is no licensed therapy for PSC proven to slow or stop disease progression, and current treatment focuses on stricture management, screening for and management of complications and assessing the need for liver transplantation. Therapy for PSC therefore represents an area of significant unmet clinical need.

Much of the focus in therapeutic development in PSC has been on drugs that modify key cholestatic pathways, with several attempts to extrapolate therapies of proven benefit in primary biliary cholangitis (PBC). Ursoedeoxycholic acid (UDCA), the first line agent used in PBC has been evaluated extensively in PSC and is sometimes used in clinical practice. However, although it has been shown to improve cholestatic serum liver blood tests, with a reduction in alkaline phosphatase, no survival benefit has been demonstrated and higher doses may lead to an increased risk of hepatic decompensation, death and need for liver transplantation [2–16]. Biochemical improvement has also been demonstrated with metronidazole [17], vancomycin [17–19], bezafibrate [20], *nor*ursodeoxycholic acid [21] and obeticholic acid [22] but, again, no proven survival benefit has been demonstrated. Until recently, current guidelines did not recommend the use of any of these agents and the identification of a specific therapy for PSC is seen as an area of the highest priority [23–25]. The 2022 European Association for the Study of the Liver (EASL) guidelines now recommend that UDCA can be used but acknowledge that the evidence for this recommendation is limited [25].

In this study, we use an *in silico* approach to identify potential novel therapy options for PSC, utilising the extensive, previously published findings regarding the genetic basis of the disease. Network Proximity Analysis (NPA) is a virtual method of exploring potential relationships between known drug targets and genes known to be associated with disease [26]. The ‘druggable genome’ uses genome-wide association study (GWAS) data alongside established drug mechanisms to catalogue possible sites of interaction. The output from this analysis approach is a list of drugs that have a genetic target known to be proximal to the disease-implicated gene, that may have an effect on genetically encoded mechanisms of disease pathogenesis. This approach, already utilised in PBC [27], allows identification of treatments that have not been previously linked to PSC, and that could be repurposed from other indications. It offers particular potential for identifying a number of candidate agents that could be systematically evaluated in an ‘adaptive’ trial model, ideally suited for rare diseases where potential trial populations are by definition limited.

## Methods

### Identification of candidate genes

**Fig. 1:**
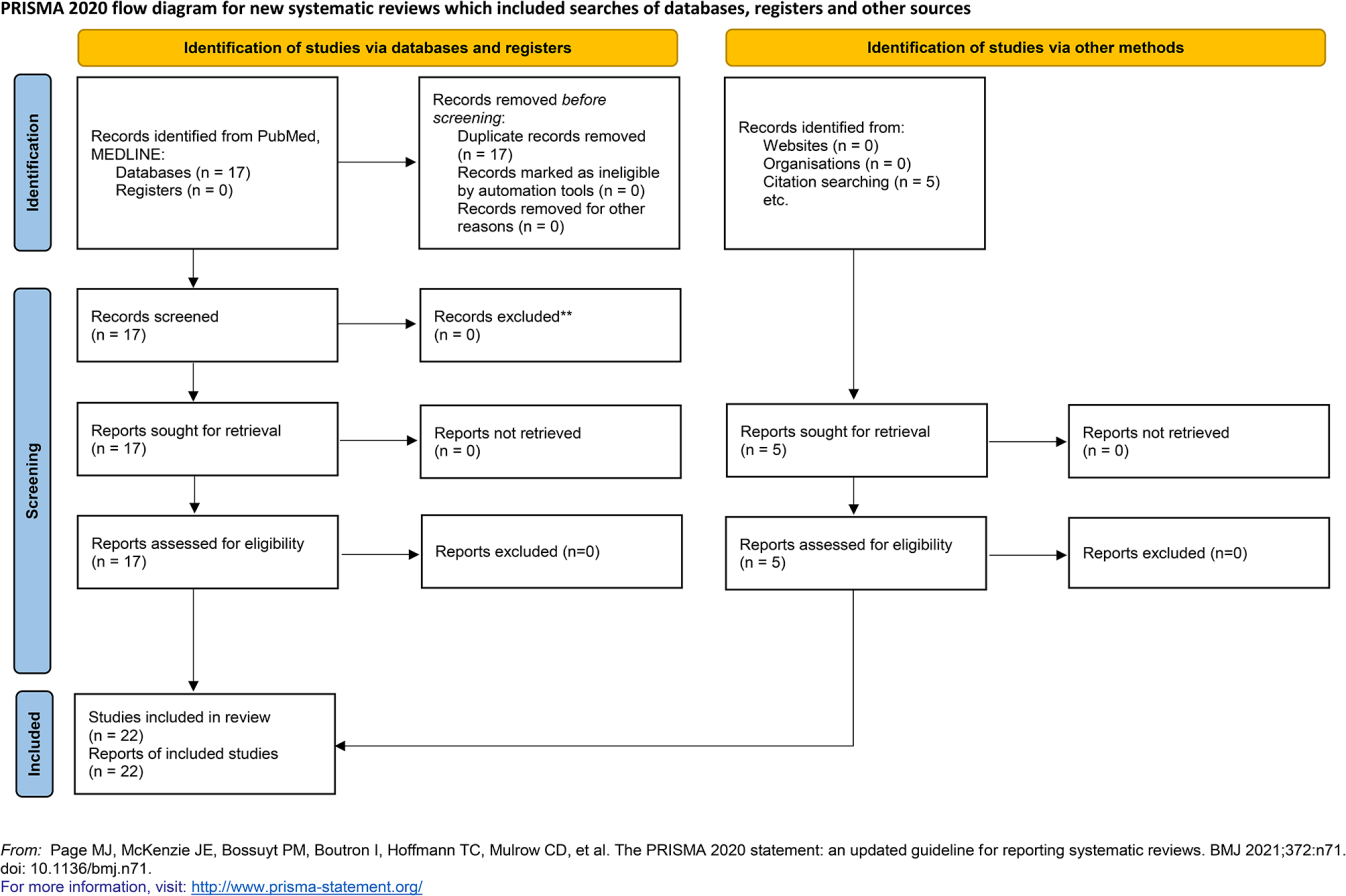
PRISMA diagram for systemic literature review

A systematic literature review was conducted in December 2020, initially searching PubMed for papers tagged with “Primary Sclerosing Cholangitis” and “GWAS”, which identified 17 full publications. On review of these publications, and additional cited publications, 22 papers were identified. These comprised 11 GWAS studies in PSC and 11 review articles or GWAS in other disease areas. This search was repeated using MEDLINE, which identified no additional papers. Clinical trials in PSC were identified from ClinicalTrials.gov [28] and from the BSG [23] and AASLD [24] guidelines to cross-reference previously investigated agents, and this list was supplemented with trials reported elsewhere in the literature. This review was conducted by a single investigator. (See Fig. 1 for PRISMA diagram).

Collation of results yielded 89 unique single nucleotide polymorphisms (SNPs) associated with PSC. Human leukocyte antigen (HLA)-associated SNPs, those that did not achieve genome-wide significance (*p*-value of < 5 × 10^− 8^) and those that did not suggest a relevant gene were excluded, leaving 26 unique genetic loci for analysis (Table 1, including duplicate association statistics) as reported in 8 studies (Supplementary Table 1).

**Table 1:** Summary of systematic literature review, identifying 26 unique genes for analysis

Suggested gene(s)	*p*-value(s)	Quoted loci and SNP
MSH5-SAPCD1 [47]	5.12E-11	6p21, rs3130484
TNFRSF14/MMEL1 [33]	2.10E-08	1p36, rs3748816
GPR35 (*2 SNPs*) [35]	3.43E-9, 2.99E-9	2q37, rs4676410 and rs3749171
TCF4 [35]	2.61E-08	18q21, rs1452787
MMEL1, TNFRSF14 [36]	7.41E-12	1p36, rs3748816
CD28 [35]	1.89E-20	2q33, rs7426056
MST1 [36]	2.45E-26	3p21, rs3197999
IL2/IL21 [36]	8.87E-13	4q27, rs13140464
BACH2 [36]	8.36E-12	6q15, rs56258221
IL2RA [36]	8.19E-17	10p15, rs4147359
SIK2 [36]	3.17E-09	11q23, rs7937682
HDAC7 [36]	5.49E-09	12q13, rs11168249
SH2B3/ATXN2 [36]	5.91E-11	12q24, rs3184504
CD226 [36]	3.06E-08	18q22, rs1788097
PRKD2/STRN4 [36]	6.51E-10	19q13, rs60652743
PSMG1 [36]	3.19E-17	21q22, rs2836883
MST1 [38]	3.80E-12	3p21, rs3197999
IL2RA (*2 SNPs*) [38]	1.5E-8, 3.4E-7	10p15, rs4147359 and rs706778
MST1 [48]	1.10E-16	3p21, rs3197999
BCL2L11 [48]	4.10E-08	2q13, rs6720394
MMEL1 [40]	5.17E-13	1, rs3748816
CD28 [40]	2.12E-16	2, rs7426056
MST1 [40]	5.11E-26	3, rs3197999
IL2, IL21 [40]	1.19E-13	4, rs13140464
BACH2 [40]	1.41E-09	6, rs56258221
IL2RA [40]	7.54E-17	10, rs56258221
SIK2 [40]	4.77E-07	11, rs7937682
SH2B3 [40]	4.27E-13	12, rs3184504
CD226 [40]	6.58E-12	18, rs3184504
PRKD2 [40]	1.99E-12	19, rs60652743
PSMG1 [40]	4.21E-13	21, rs2836883
BCL2L11 [40]	2.36E-11	2, rs72837826
FOXP1 [40]	2.62E-15	3, rs80060485
CCDC88B [40]	2.24E-13	11, rs663743
CLEC16A [40]	3.59E-13	16, rs725613
UBASH3A [40]	2.19E-12	21, rs1893592
NFKB1 [41]	3.81E-10	4, rs17032705
RIC8B [41]	1.29E-09	12, rs12369214

### Network proximity analysis

**Fig. 2:**
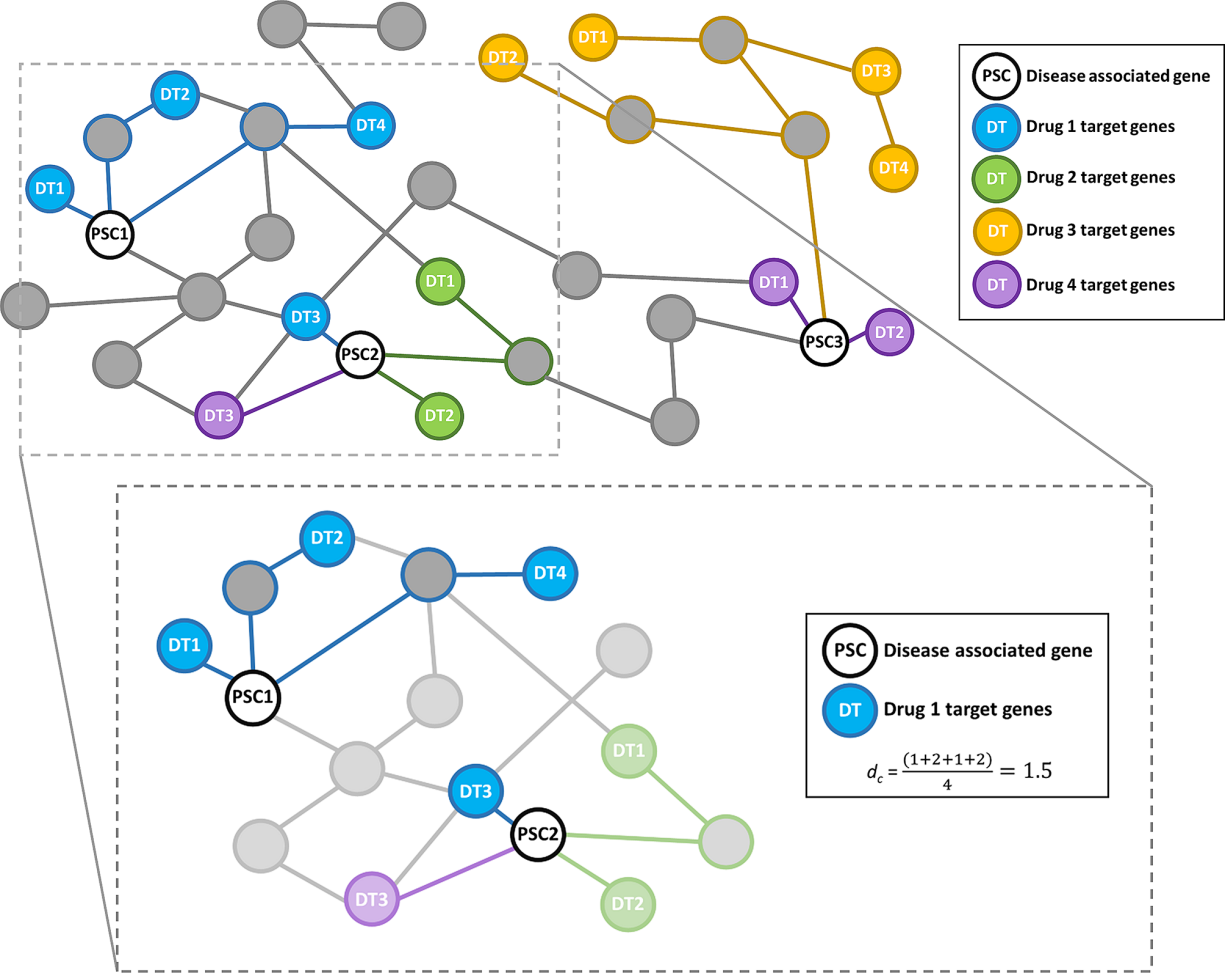
For each drug, the known target genes (nodes of the same colour) are linked to their nearest disease-associated genes (white nodes with black edging) to calculate the “distance” *d*_*c*_ between the drug and the disease. For Drug 1 (blue), the distance is the average of the four blue pathways (the distances from each of the drug target genes to the nearest disease associated gene) i.e. *d*_*c*_ = 1.5. Drug 2 (green) has only two target genes but the same *d*_*c*_ = 1.5. Drug 3 (orange) has four target genes which are all quite distal and has *d*_*c*_ = 3.25. Drug 4 (purple) has two drug target genes closest to PSC3 and one closest to PSC2, with an overall *d*_*c*_ = 1. The final relative proximity measure z between each drug and the disease is calculated as *z=(d*_*c*_*- µ)/σ *where *µ* and *σ* are calculated empirically via a randomisation procedure

This study used the Python code [29] and drug-disease network validated by Guney et al. [30] to seek drug targets for the candidate genes. The method uses a previously published interactome network [31] and has demonstrated that drug target-disease proximity is a good marker of efficacy. As illustrated in Fig. 2, for each drug, the method calculates *d*_*c*_ (the average of the distances to the closest disease associated gene for each drug target gene) and this is used to calculate a z-score (*z=(d*_*c*_*- µ)/σ*) using a randomisation procedure to empirically calculate *µ* and *σ*. The z-score end result is a score of drug-disease proximity for each of the drugs from the DrugBank [32] resource (a freely available drug database containing known genetic drug targets) (February 2021 version). Guney et al. [30] validated a cut-off for z-score of ≤ -0.15 to infer that the drug is proximal to the disease and may exert a pharmacological effect, based on known drug-disease effects. In order to identify compounds most strongly associated with PSC implicated pathways, and therefore that may be most clinically relevant, we chose to use a more stringent cut-off z score of -2.0.

The methodology used here was purely a secondary analysis of published data from previous studies, and did not involve any direct patient information. As such, no ethical approval was required. Previously published data about NPA in PBC was utilised in this study as a comparator for PSC [27] rather than collection of new PBC data.

## Results

Network proximity analysis of 6296 compounds identified 2528 compounds with z scores ≤ -0.15 and 101 with z scores ≤ -2.0 for PSC (Supplementary Table 3), many of which are not medicinal products. Given that the focus of this study was to identify plausible candidate therapies, non-medicinal compounds were not considered further. A total of 42 medicinal products potentially appropriate for systemic therapeutic use showed a z score of ≤ -2.0 (Table 2). Of those, 23 are already licensed for another indication and therefore may be candidates for repurposing in PSC (denoted by * in the table). Only one identified compound (metronidazole) has, to our knowledge, been suggested as a potential therapy for PSC.

**Table 2:** Medicinal compounds with z ≤ -2.0 in PSC (*n* = 42), (* denotes already licensed)

z	Mean	sd	Drug	Relevant gene and direction of relationship, bold indicating a likely therapeutic effect	Indications
-5.08699	1.992667	0.260665	Denileukin diftitox*	IL2RB_target_agonist IL2RG_target_ IL2RA_target_binder	Cutaneous T-cell lymphoma
-5.03786	1.9735	0.292485	Basiliximab*	IL2RA_target_**antibody** IL2RB_target_**antibody**	Kidney transplant rejection prophylaxis
-3.78712	2.4515	0.383272	Abatacept*	CD86_target_**antagonist** CD80_target_**antagonist**	Juvenile idiopathic and rheumatoid arthritis
-3.73261	2.4435	0.386727	Belatacept*	CD80_target_**antagonist** CD86_target_**antagonist**	Kidney transplant rejection prophylaxis
-3.63858	2.317	0.361954	Girentuximab	IL2_target_ CA9_target_	Investigated in gallbladder and renal cell cancer
-3.51662	1.938333	0.266828	Navitoclax	BCL2L2_target_ BAD_target_ BCL2_target_	Investigated in multiple cancers
-3.11562	1.790667	0.253775	Isosorbide*	MCL1_target_ BCL2L1_target_ BCL2_target_	Angina
-3.03871	2.185	0.280273	Tapinarof	IL2_target_ IL12B_target_ IL6_target_	Investigated in psoriasis
-2.9965	1.9765	0.32588	TG4010	IL2_target_ MUC1_target_	Investigated in cancers
-2.75936	1.391	0.504102	NF-kappaB Decoy	NFKB1_target_**inhibitor**	Investigated in inflammatory disorders
-2.73881	1.963	0.351612	KD3010	PPARD_target_	Investigated in metabolic disease and obesity
-2.73051	2.00725	0.117103	CYT997	TUBB1_target_ TUBB2B_target_ TUBA1A_target_ TUBB2A_target_ TUBA1C_target_ TUBA4A_target_ TUBA3E_target_ TUBB6_target_ TUBB4B_target_ TUBA3C_target_ TUBA3C_target_ TUBB3_target_ TUBB4A_target_ TUBA4B_target_ TUBB_target_ TUBA1B_target_	Investigated in solid tumours
-2.71394	2.486	0.547544	Galiximab	CD80_target_**antibody**	Investigated in lymphoma, canceers, rheumatoid arthritis
-2.64154	1.448	0.358882	P54	NFKB2_target_ NFKB1_target_	Investigated in cancers, IBD, OA
-2.63165	2.301	0.494367	SPP 301	EDNRA_target_	Investigated in cardiovascular disorder and diabetic neuropathy
-2.57539	2.271	0.493517	Darusentan*	EDNRA_target_	Heart failure, hypertension
-2.57238	2.28	0.497594	Actelion-1	EDNRA_target_	Investigated in cardiovascular disorder, hypertension, pulmonary hypertension
-2.56224	1.4565	0.373307	SGN-30	NFKB2_target_ NFKB1_target_	Investigated in autoimmune disease, cancers
-2.5602	1.449	0.370674	NOX-700	NFKB2_target_ NFKB1_target_	Investigated in T2DM
-2.54378	2.326	0.521272	Ecallantide*	KLKB1_target_**inhibitor**	Hereditary angioedema
-2.51841	2.259	0.499919	Atrasentan	EDNRA_target_	Investigated in cancers
-2.51459	1.436667	0.306213	Custirsen	NFKB2_target_ ESR1_target_ NFKB1_target_	Investigated in brain/breast cancers
-2.51227	2.095	0.435861	Propyl alcohol*	LYZ_target_	Skin disinfection
-2.50417	2.272	0.507953	Clazosentan	EDNRA_target_	Investigated in stroke
-2.47432	1.695667	0.281155	HE3286*	NFKB2_target_ CYP3A4_enzyme_substrate NFKB1_target_	T2DM and RA
-2.47009	1.948	0.383792	Thiocolchicoside*	GLRA1_target_**antagonist** TNFSF11_target_**antagonist**	Back pain, osteoarthritis, rheumatoid arthritis
-2.46616	2.304	0.528757	Lanadelumab*	KLKB1_target_**inhibitor**	Hereditary angioedema, angioedema
-2.41208	2.088189	0.042463	Fostamatinib*	*Over 300 genes and targets, see supplementary Table* 6	Chronic immune thrombocytopoenia
-2.32862	2.392	0.239913	Cefazolin*	IL15_target_**inhibitor** SLC22A8_transporter_**inhibitor** ALB_carrier_other/unknown SLC22A11_transporter_**inhibitor** ABCC4_transporter_substrate PON1_target_**inhibitor** IL2_target_**inhibitor** SLC22A6_transporter_substrate|**inhibitor** TPMT_enzyme_substrate	Infections
-2.32062	1.937	0.403771	Denosumab*	TNFSF11_target_**antibody**	Osteoporosis
-2.28665	2.289389	0.126556	Promethazine*	HRH1_target_**antagonist** ABCB1_transporter_**inhibitor** CHRM2_target_**antagonist** P2RY10_target_**inhibitor** DRD2_target_**antagonist** HRH2_target_**antagonist** CHRM1_target_**antagonist** SCN9A_target_**inhibitor** CYP2D6_enzyme_substrate|**inhibitor** CYP2D6_enzyme_substrate|**inhibito** ABCC3_transporter_**inhibitor** CHRM5_target_**antagonist** KCNAB2_target_inducer ABCC4_transporter_**inhibitor** CYP2C9_enzyme_**inhibitor** CYP2B6_enzyme_substrate ALB_carrier_binder CALM1_target_**inhibitor** CALM1_target_**inhibitor** CALM1_target_**inhibitor** ADRA2C_target_**antagonist** CHRM3_target_**antagonist** CHRM4_target_**antagonist**	Allergy
-2.25564	2.3155	0.361538	Tezosentan	EDNRA_target_ EDNRB_target_	Investigated in heart failure, liver disease, heart disease
-2.25489	1.95	0.421307	AMGN-0007	TNFSF11_target_	Investigated in osteoporosis and bone mets
-2.16756	2.304	0.370923	Enrasentan	EDNRA_target_ EDNRB_target_	Investigated in heart failure, COPD, BPH
-2.13883	2.291	0.291904	Bictegravir*	POU2F2_enzyme_**inhibitor** CYP3A4_enzyme_substrate SLC47A1_enzyme_**inhibitor** UGT1A1_enzyme_substrate	HIV
-2.11312	1.8174	0.197528	Andrographolide	NFKB1_target_ IL6_target_TNF_target_ NFKB2_target_ IL1B_target_	Investigated in UC
-2.10096	2.438	0.208476	Metronidazole*	ABCB1_transporter_**inhibitor** CYP3A4_enzyme_**inhibitor** CYP3A5_enzyme_substrate CYP2A6_enzyme_substrate CYP3A7_enzyme_substrate CYP3A7_enzyme_substrate CYP2C9_enzyme_**inhibitor** UGT1A1_enzyme_substrate CYP2C8_enzyme_**inhibitor**	Infections
-2.03487	1.979214	0.130194	Pseudoephedrine*	ADRB2_target_partial agonist MAOA_enzyme_**inhibitor** TNF_target_**inhibitor** ADRA2A_target_agonist NFKB1_target_**inhibitor** SLC6A4_transporter_**inhibitor** SLC6A3_transporter_**inhibitor** NFATC1_target_**inhibitor** JUN_target_**inhibitor** ADRB1_target_agonist|partial agonist IL2_target_**inhibitor** ALB_carrier_binder ADRA1A_target_agonist SLC6A2_transporter_**inhibitor**	Allergy, congestion
-2.02175	1.856	0.423396	Ancestim*	KIT_target_agonist	Stem cell harvest
-2.01682	2.102	0.29849	Castor oil*	PTGER4_target_agonist PTGER3_target_agonist|activator	Constipation
-2.01298	2.262714	0.201478	Tucatinib*	ERBB3_target_**inhibitor** SLC22A2_transporter_**inhibitor** CYP3A7_enzyme_substrate CYP3A7_enzyme_substrate CYP2C8_enzyme_substrate ABCB1_transporter_substrate ERBB2_enzyme_**inhibitor** SLC47A2_transporter_**inhibitor** SLC47A1_transporter_**inhibitor** ABCG2_transporter_substrate	Breast cancer
-2.01138	2.182278	0.118244	Pazopanib*	FGFR3_target_**inhibitor** FLT4_target_ PDGFRB_target_**inhibitor** ITK_target_**inhibitor** SH2B3_target_**inhibitor** CYP1A2_enzyme_substrate ABCB1_transporter_substrate SLCO1B1_transporter_**inhibitor** **UGT1A1_transporter_inhibitor** KIT_target_**inhibitor** CYP2C8_enzyme_substrate|**inhibitor** CYP2D6_enzyme_**inhibitor** CYP2D6_enzyme_**inhibitor** ABCG2_transporter_substrate KDR_target_**inhibitor** CYP3A4_enzyme_substrate|**inhibitor** FLT1_target_**inhibitor** PDGFRA_target_**inhibitor** FGF1_target_**inhibitor**	Renal cell, soft tissue, thyroid cancer

The agents already in clinical use for other indications with the lowest z scores, indicating very close proximity to a disease associated gene, are all immune modulators; Denileukin diftitox (-5.087), Basiliximab (-5.038), Abatacept (-3.787) and Balatacept (-3.73). Isosorbide, used in angina, was the only non-immunomodulatory agent with a highly proximal z-score (-3.116).

Table 3 lists the proximities of drugs currently or previously trialled in PSC (as recorded on ClinicalTrials.gov [28] or with published data) to evaluate whether they would have been identified as plausible candidates using NPA methodology i.e. likely to have an effect on the genetically encoded pathogenesis of PSC. There were 11 compounds with a z score ≤ -0.15 but only metronidazole had a z score ≤ -2.0.

**Table 3:** **Proximity scores for drugs trialled in PSC and trial details**

Drug (clinicaltrials.gov identifier or paper reference)	z-value	Trial result details
Metronidazole (NCT01085760)	-2.100964165	*n* = 18, 77% IBD, split between high and low dose, demonstrated efficacy but didn’t meet primary outcome [17]
Volixibat (NCT04663308)	-0.97191795	Ongoing
Vancomycin (NCT03710122, NCT02605213, NCT01802073/NCT01322386, NCT01085760, NCT02137668, NCT03046901, Damman et al. 2018)	-0.96744415	Recruiting Recruiting *n* = 59, ages 1.5–44, 95% IBD, biochemical improvement (normalisation in 22–55%)[18] *n* = 17, 65% IBD, split high and low dose, significant improvement in both [17] Recruiting Withdrawn *N* = 98 various trials, insufficient evidence [19]
Budesonide (NCT00004842)	-0.821600362	Completed - no report
Cladribine (NCT00004762)	-0.794671207	Completed - no report
Fenofibrate (NCT01142323)	-0.644737496	Terminated - no report
Erlotinib - for cholangiocarcinoma prevention in trisomy 7 (NCT00955149)	-0.575691734	Completed - no report
Docosahexaenoid Acid – DHA, doconexent (NCT00325013)	-0.467860521	Completed - no report
Bezafibrate (NCT04309773, NCT02701166, Mizuno et al. 2015)	-0.434430331	Studies recruiting/in progress *N* = 15, 11 weeks, biochemical improvement [20]
Minocycline (NCT00630942)	-0.363246802	Completed - no report
All-trans Retinoic Acid – tretinoin (NCT03359174, NCT01456468)	-0.254372936	Terminated - no report Reduced liver fibrosis (better with UDCA) in bile duct ligated rats [16]
Vedolizumab (NCT03035058)	0.027695758	Withdrawn - no report
Xifaxan - rifaximin (NCT01695174)	0.352010243	*n* = 16, 81% IBD, no significant change in biochemistry or symptoms [49]
Sulfasalazine (NCT03561584)	0.68636947	Recruiting
Simvastatin (NCT04133792)	0.880045656	Recruiting
Ursodeoxycholic acid (NCT01088607, NCT00059202, NCT01456468, Beuers et al. 1992, Chazouillères et al. 1990, O’Brien et al. 1991, Lindor et al. 1997, Olsson et al. 2005, Lindor et al. 2009, De Maria et al. 1996, Charatcharoenwitthaya et al. 2007, Wunsch et al. 2014, Mitchell et al. 2001, van Hoogstraten et al. 1998, Stiehl et al. 1994, Cullen et al. 2008, Harnois et al. 2001,)	1.041833924	Completed - no report Completed - no report Reduced liver fibrosis (better with atRA) in bile duct ligated rats [16] *n* = 14 vs. placebo, improvement in biochemistry and histology [2] *n* = 15, biochemical and clinical improvement [3] *n* = 12, biochemical improvement in treatment periods, deterioration on withdrawal [4] *n* = 105 vs. placebo, biochemical improvement but no benefit in time to treatment failure [5] *n* = 219 vs. placebo, non-significant biochemical difference, no clinical outcome difference [6] *n* = 150 vs. placebo, biochemical improvement, no survival improvement, serious adverse events [7] *n* = 59 vs. colchicine vs. control, no long term benefit [8] *n* = 42 (+/- IBD, 37 given UDCA, biochemical improvement but no change in outcome [9] *n* = 26, UDCA withdrawal, worsening of biochemistry and pruritus after 3 months [10] *n* = 26 vs. placebo, biochemical, histological and cholangiographic improvement at 2yrs [11] *n* = 48 vs. placebo, biochemical improvement but no difference for symptoms or histology [12] *n* = 24 induction then half vs. placebo, significant biochemical improvement, not in symptoms [13] *n* = 31 low/standard/high dose, biochemical improvement, prognostic benefit in high only [14] *n* = 30, compared with previous study control, biochemical improvement at 1yr [15]
Curcumin (NCT02978339)	1.108613924	*n* = 15, 20% met primary outcome
Obeticholic acid (NCT02177136)	1.170058504	*n* = 76, IBD ~ 6-%, ALP significantly reduced in 5-10 mg/day, pruritis in 60–67% of treatment groups [22]
Thalidomide (NCT00953615)	1.294318063	Terminated - lack of enrolment
Mitomycin C (NCT01688024)	1.513646452	Recruiting

Given the strong relationship between PSC and inflammatory bowel disease (IBD), we explored the proximity in NPA for PSC of agents that are already established in IBD therapeutics (Table 4). Corticosteroids were significantly proximal with budesonide having a z-score of -0.822 and prednisolone (in its various forms) having z-scores of ≤ -0.15. However, the other currently available treatments were not proximal, all having positive z scores. Ozanimod, a sphingosine-1-phosphate receptor modulator, had a z score of -0.202 but remains in trial phase and is not currently licensed. It is important to note, however, that of the PSC studies in which the significant SNPs were identified, there was considerable heterogeneity in terms of comorbid IBD [33–41]. In time, with further characterisation of the PSC-IBD phenotype and genotype, these groups may need to be stratified for further genetic studies.

**Table 4:** PSC z scores for drugs used in inflammatory bowel disease

Drug	z score
Prednisone	-0.97011
Budesonide	-0.822
Prednisolone	-0.6956
Ozanimod	-0.202
Mesalazine	0.003
Vedolizumab	0.028
Cyclosporine	0.06
Ustekinumab	0.17
Adalimumab	0.27
Golimumab	0.281
Infliximab	0.335
Sulfasalazine	0.686
Tofacitinib	0.851
Tofacitinib	0.851
Mercaptopurine	1.227
Methotrexate	1.369
Azathioprine	1.602
Certolizumab	1.622

Therapies have previously been extrapolated from PBC to PSC without success in terms of demonstrating survival benefit. When these NPA methods were applied in PBC [27], published data showed 2637 compounds with z values ≤ -0.15 identified and 253 with a z score ≤ -2.0. Of those with a z score ≤ -2.0, 109 were medicinal compounds. None of the therapies with confirmatory evidence of benefit from clinical trials in PBC had a z score of ≤ -2.0 (UDCA 0.171, obeticholic acid − 0.737. bezafibrate − 0.866, fenofibrate − 0.986) and UDCA did not meet the minimal threshold of significant proximity of -0.15, so was not identified as a proximal compound.

Table 5 lists the 20 candidates achieving significant proximity with a z score of ≤ -2.0 in both PBC and PSC that are already in use for another indication or under investigation. Supplementary Table 5 provides a full list of all compounds with a z score of ≤ -2.0 or better in either or both diseases. We again observe the biological agents seen earlier (Basiliximab, Balatacept, Abatacept, Denileukin diftitox) and a number of compounds utilised or under investigation in other immune-mediated diseases (psoriasis, inflammatory bowel disease, rheumatoid arthritis). The analysis identified non-biological agents that are proximal in both diseases (for example, the retinoids Arotinoid acid and Acitretin).

**Table 5:** Compounds with z score ≤-2.0 for both PSC and PSC with current use or under investigation

Drug	PSC z-score	PBC z-score	Current use	Under investigation for
Denileukin diftitox	-5.087	-3.975	Cutaneous T-cell lymphoma	
Basiliximab	-5.038	-3.320	Kidney transplant rejection prophylaxis	
Abatacept	-3.787	-4.603	RA/psoriatic arthritis/GVHD prophylaxis	
Belatacept	-3.733	-4.709	Kidney transplant rejection prophylaxis	
NF-kappaB Decoy	-2.759	-2.809		inflammatory disorders
CYT997	-2.731	-2.593		solid tumours
Galiximab	-2.714	-4.217		lymphoma, psoriasis, RA
P54	-2.642	-2.362		Cancers, IBD, OA
Arotinoid acid	-2.637	-4.419		psoriasis
SGN-30	-2.562	-2.401		autoimmune disease, cancers
NOX-700	-2.560	-2.461		T2DM
Custirsen	-2.515	-2.206		brain/breast cancers
HE3286	-2.474	-2.163	T2DM and RA	
Thiocolchicoside	-2.470	-4.217		rheumatological disorders, muscle contraction
Fostamatinib	-2.412	-3.305	Chronic ITP	
Denosumab	-2.321	-4.416	Osteoporosis	
AMGN-0007	-2.255	-4.203		osteoporosis/bone metastases
Andrographolide	-2.113	-2.592		UC
Tucatinib	-2.013	-2.821	Breast cancer	
Pazopanib	-2.011	-2.462	Renal cell, soft tissue, thyroid cancer	

## Discussion

In this *“in silico*” study we set out to use network proximity analysis to identify previously un-heralded candidate therapies for potential clinical evaluation in PSC, based on their likelihood of action on genetically-identified causative disease pathways. To our knowledge, this is the first time this approach has been used in PSC. The approach has been applied to a number of other chronic disease areas, including PBC [42], and has been proposed as a hypothesis-free methodology for identifying potentially valuable, novel approaches to therapy in disease areas with unmet clinical need.

While this method is known to demonstrate meaningful associations, it is important to note there is not necessarily implied directionality (drugs effective at these loci may worsen rather than improve the pathology), nor any guarantee the GWAS-identified genes are truly implicated, rather than associated due to linkage disequilibrium or a non-coding transcription regulation region. However, given the availability of the GWAS data and validation of this method in other disease areas, the method is certainly an appropriate source of potential candidate drugs in PSC therapy. Also included in Table 2 are the gene target descriptions for the associated drugs, with those known to be inhibitors/antagonists highlighted as likely to down-regulate expression of the implicated genes (of note, this would need to be further investigated prior to any clinical trials, as reducing expression of a regulatory gene, for example, may exacerbate disease).

With no currently licensed therapy, PSC is a disease with obviously unmet clinical need. It is also a rare and heterogeneous disease meaning that there are limited numbers of patients who can be recruited into clinical trials and the number of trials that can be conducted at any one time is restricted. This gives rise to “opportunity cost” in terms of less promising trials utilising the available patient pool and, as a result, preventing other trials of potentially more promising agents from being conducted. PSC is a condition in which novel potential therapeutic approaches are needed, in order to prioritise selection of agents for incorporation into trials. Network Proximity Analysis (NPA) is an approach that could potentially provide a solution to both of these challenges. By assessing the degree to which genetically encoded disease pathways showing a significant association with PSC co-map to predicted drug actions, NPA allows us to identify drugs that show a significant likely association with a disease-related pathway and which could therefore be novel candidate therapies. The converse is also true in that a drug with no apparent mechanistic effect on any PSC disease pathway might be less likely to be effective and thus a lower priority for trial evaluation. Using this approach, we identified a number of drugs with PSC pathway associations, and thus candidate therapies. The majority of these have not previously been identified as potential therapies for PSC.

The already licensed (and thus most suited to repurposing) drugs with the strongest predicted pathway association with PSC are biological immune-modulatory therapies. These include the IL-2R (CD25) monoclonal antibody Basiliximab and Balatacept and Abatacept that block CD80/CD86 blocker interaction with T-cells. The strongest candidate drug of all in terms of degree of association is denileukin diftitox (an IL2 binding cytocidal agent) that is currently used in cutaneous T-cell lymphoma, although its action on IL2 is agonistsic/binding.

When degree of proximity was assessed for therapies already trialled in PSC there was a striking lack of association. The only agent showing a z-score < -2.0 (a strong association, albeit markedly weaker than the drugs outlined above) was metronidazole. This drug has been shown to give modest improvement in liver biochemistry in a combination study with Ursodeoxycholic Acid [43]. The 2004 study of 80 patients randomised to either metronidazole or placebo in combination with UDCA showed improvement of liver biochemistry in both groups, with alkaline phosphatase (ALP) significantly more reduced in the metronidazole group (*p* < 0.05) after 36 months (although there was no significant impact on disease progression or long-term follow up). Weaker, but still significant, proximity was seen for Vancomycin (-0.967) and Bezafibrate (-0.434); drugs shown to have some benefit in terms of biochemical improvement in PSC [17–20]. In contrast, UDCA (widely used in PSC although with no confirmed evidence of survival benefit) and Obeticholic Acid (OCA; a licensed second-line therapy in PBC and previously trialled in PSC) were not significantly proximal with z scores of 1.042 and 1.170, respectively.

Comparison of network proximity for PSC and PBC, diseases that have clinical features in common and in which overlapping therapy approaches have been explored (with varying degrees of success), shows interesting similarities and differences in the potential therapies identified. Medications previously trialled in PSC (including UDCA and OCA) that have been ‘borrowed’ from PBC for their effects on cholestasis appear to have no genome-level basis to their effect, potentially highlighting cholestasis as a common end-pathway to two different pathologies. Overall, PBC NPA identifies more candidate agents than PSC, including whole classes of drugs, such as the kinase inhibitors that are strong candidates in PBC. The approach does identify a number of un-anticipated agents that are candidates in either PSC alone or in both PBC and PSC (exemplified by isosorbide in PSC) that could be systematically evaluated in a repurposing-focused advanced trial model. There are ongoing trials examining new agents for use in PSC that are not yet part of DrugBank (such as nor-ursodexoycholic acid [21], cenicriviroc [44] and vidofludimus [45] calcium) so were not included in this study. This means the results of this analysis will be dynamic as both the drug-disease network and the DrugBank resource are updated and refined.

Where, if anywhere, does the analysis presented here leave us with regard to PSC therapy? The approach is a seductive one; generating an intriguing list of drugs that we could evaluate in what is currently an untreatable disease. It is one, however, with a number of important caveats. The first caveat is that although the approach has been taken in a number of disease areas (including PBC), the result has been the same each time; a list of interesting drugs but no progress beyond that. The next step is to incorporate these candidate drugs into a real-world clinical trial, and to formally test the hypothesis that the NPA approach *in silico* identifies drugs that have actual therapeutic effects in the target disease in clinical trials. Until this happens the approach remains an interesting side-line. The second caveat about the NPA approach is that whilst it shows a relationship between a molecule’s modes of action and a disease pathway, it does not tell us the direction of that relationship clinically. It is conceivable that the approach identifies a drug that actually worsens rather than improves a disease. This needs to be remembered (and ideally explored theoretically prior to implementation) if and when we move from this analysis to a clinical trial. The third caveat is that by its nature the approach only addresses the genetic component of a disease. PSC, in common with most chronic inflammatory diseases, has both a genetic and an environmental component to its aetiology. However, as demonstrated in PBC (UDCA is the mainstay of treatment with proven benefit, but a non-proximal z-score of 0.171), non-proximity at a genome level does not rule out drug efficacy and NPA is not a method to retrospectively validate treatments. Any therapy that would work on an environmental component will not be flagged up using this analysis approach. An important example might be a therapy modulating the microbiome in PSC. In this regard it is interesting that metronidazole and vancomycin are flagged up and yet might be expected to work on the environmental arm of aetiology. Their identification as candidates through NPA raises the interesting possibility that their mode of action might be unrelated to their anti-microbial actions. The final caveat is that the approach may identify a candidate drug but it does not tell you how and when to use it. This is exemplified by Ustekinumab in PBC; a very strong candidate targeting a disease pathway strongly associated with PBC. The clinical trial of the drug in PBC was, however, negative [46]. One explanation for this apparent paradox would be that NPA in fact doesn’t reliably identify candidate therapies. The alternative might be that the Ustekinumab trial targeted people who had failed UDCA therapy (i.e. people with “downstream”, cholestasis-driven disease rather than disease in an “upstream”, immune stage). Given the immune-modulatory nature of almost all of the drugs identified for PSC in this analysis, the lessons of the PBC Ustekinumab experience for future trial design in PSC are clear.

While there are important caveats to remember, this method has identified drugs with known safety profiles that would be potential candidates for trials in PSC; a disease with otherwise no effective therapeutic options. This *in silico* exploration of therapeutics is a safe and novel way to identify candidate drugs to optimise efforts in rare disease trials and would form a helpful basis for further research into the use of metronidazole or initially use of biologics such as basiliximab, abatacept or belatacept.

### Electronic supplementary material

Below is the link to the electronic supplementary material.

Supplementary Material 1

Supplementary Material 2

Supplementary Material 3

## Data Availability

All data generated or analysed during this study are included in this published article and its supplementary information.
