# Supplementary material for: Network proximity analysis as a theoretical model for identifying potential novel therapies in primary sclerosing cholangitis
Source: BMC Med Genomics. 2024 Jun 11;17:157. doi: 10.1186/s12920-024-01927-2 (PMC11165726; doi:10.1186/s12920-024-01927-2)
Supplement: Supplementary file 1 — Supplementary Material 1 [file 12920_2024_1927_MOESM1_ESM.docx]

**Identification of studies via other methods**

**Identification of studies via databases and registers**

Records identified from:

Websites (n = 0)

Organisations (n = 0)

Citation searching (n = 5)

etc.

Records removed *before screening*:

Duplicate records removed (n = 17)

Records marked as ineligible by automation tools (n = 0)

Records removed for other reasons (n = 0)

Records identified from PubMed, MEDLINE:

Databases (n = 17)

Registers (n = 0)

**Identification**

Records screened

(n = 17)

Records excluded**

(n = 0)

Reports not retrieved

(n = 0)

Reports sought for retrieval

(n = 5)

Reports sought for retrieval

(n = 17)

Reports not retrieved

(n = 0)

**Screening**

Reports excluded (n=0)

Reports excluded (n=0)

Reports assessed for eligibility

(n = 5)

Reports assessed for eligibility

(n = 17)

Studies included in review

(n = 22)

Reports of included studies

(n = 22)

**Included**

*From:*  Page MJ, McKenzie JE, Bossuyt PM, Boutron I, Hoffmann TC, Mulrow CD, et al. The PRISMA 2020 statement: an updated guideline for reporting systematic reviews. BMJ 2021;372:n71. doi: 10.1136/bmj.n71.
For more information, visit: <http://www.prisma-statement.org/>
